# Influence of Leader Mindfulness on the Emotional Exhaustion of University Teachers: Resources Crossover Effect

**DOI:** 10.3389/fpsyg.2021.597208

**Published:** 2021-02-24

**Authors:** Beini Liu, Zehui Zhang, Qiang Lu

**Affiliations:** ^1^School of Business, Beijing Technology and Business University, Beijing, China; ^2^School of E-Business and Logistics, Beijing Technology and Business University, Beijing, China

**Keywords:** leader mindfulness, workplace telepressure, self-efficacy in managing negative emotions, university teacher mindfulness, emotional exhaustion

## Abstract

This study combined conservation of resources theory with the job demands-resources model to explore the influence of leader mindfulness on the emotional exhaustion of university teachers Using a time-lagged research design, 388 paired data sets were gathered. Multiple regression and bootstrapping were used to test each hypothesis. The results showed that first, leader mindfulness significantly reduces the emotional exhaustion of university teachers. Second, the results showed that workplace telepressure partially mediates the relationship between leader mindfulness and the emotional exhaustion of university teachers. Third, university teacher mindfulness positively moderates the relationship between leader mindfulness and workplace telepressure. Finally, the results of this study indicate that self-efficacy in managing negative emotions negatively moderates the relationship between workplace telepressure and the emotional exhaustion of university teachers. This study empirically examined the interpersonal influence of leader mindfulness and the initial resources effect of university teacher mindfulness and self-efficacy in managing negative emotions from the bilateral perspective of leaders and university teachers.

## Introduction

Due to the introduction of the new managerialism to the field of higher education, practices such as performance orientation, project driving, and the tournament theory promotion system have changed the professional ecology of university teachers (Watts and Robertson, 2011). The working hours of university teachers have continuously increased (Yin et al., 2017) and occupational stress has become a significant characteristic of the professional life of teachers (Han et al., 2019; Yin et al., 2020). In 2018, the Central Committee of the Communist Party of China (CPC) issued “Opinions on Comprehensively Deepening the Reform of the Construction of Teachers in the New Era.” This is the first policy document issued by the Central Committee of the CPC for teachers since the founding of New China in 1949. This document clearly stated that “teachers should be concerned about their physical and mental health and overcome job burnout.” Emotional exhaustion has been recognized as the key component of job burnout, and it is a stress response caused by excessive mental exertion and extreme fatigue under pressure (Watkins et al., 2014).

Conservation of resources (COR) theory provides a theoretical perspective for understanding the processes underlying the stress response. Previous studies have focused on individuals’ efforts to acquire, maintain, cultivate, and protect resources that they value (Hobfoll, 2011). Therefore, scholars have proposed that the interpersonal transmission process of resources should be investigated by applying a resource crossover effect perspective (Hobfoll et al., 2018). Since leaders occupy higher positions in the workplace and control more resources, they can be regarded as credible sources for the outflow of resources (Rogers and Ashforth, 2017). Within the university context, “leaders” refer to managers who have decision-making responsibilities in universities and are primarily responsible for managing various activities such as budget management, inspecting student achievements, and teacher effectiveness (Caryn, 2015). In China, leaders within the university context include academic and administrative leaders at all levels, such as the university president, provost, deans, and department chairs.

Mindfulness originates from eastern Buddhist thought and emphasizes observing the current moment without judgment of attitudes or cognitive distortions (Kabat-Zinn, 2003). The concept of mindfulness has been applied to clinical medical research (Baer, 2003) and was later introduced to the field of occupational health psychology, where mindfulness has been considered to be a stable mood or tendency trait (Dane and Brummel, 2014). In the last 5 years, with the growth of positive psychology, mindfulness has gradually been introduced to the field of education (Caryn, 2015). Studies have been published on “mindfulness in teaching,” based on classroom management (Frank et al., 2015), and “mindfulness agency in learning,” based on lifelong learning ability (Deakin et al., 2015). As a combined concept of management and education, a field of research in educational management theories on mindfulness in university leaders has gradually emerged (Caryn, 2015).

Most existing research on mindfulness in the workplace has focused on how the intrapersonal effects of mindfulness benefit the individual (Sutcliffe et al., 2016). However, research that examines the interpersonal aspect of leader mindfulness on subordinates is relatively lacking (Good et al., 2016). Because of leaders’ hierarchical position and prestige, their attitudes and behaviors exert considerable influence on employees’ own attitudes and behaviors (Rogers and Ashforth, 2017). Scholars and practitioners have suggested that mindfulness may positively affect the behavior of leaders, thus affecting employee outcomes (Baron et al., 2018). Research into mindfulness is increasing exponentially, but specific research into leader mindfulness is still unfocused (Decuypere et al., 2020), and empirical evidence is scarce (Schuh et al., 2019). As a result, several scholars have called for a stronger focus on the interpersonal effects of leader mindfulness among employees (Reb et al., 2019). Therefore, based on COR theory, the first purpose of this study is to test the influence of university leader mindfulness on the emotional exhaustion of university teachers from the perspective of the resources crossover effect.

With the continuous and extensive application of mobile communication technology, organizations increasingly rely on asynchronous communication tools to foster a continuous connection between employees and their work (Butts et al., 2015). These information and communication technologies (ICTs) make employees more accessible during non-working hours, which can better promote the establishment of employee relationship networks and thus improve work performance (Ou et al., 2013). However, the application of mobile communication technology also has several negative effects. The use of asynchronous communication tools makes it challenging for employees to be detached from work during non-working hours, resulting in work-family conflicts and the detrimental interference of an employees’ occupation with their life (Barber et al., 2019). This kind of work-on-demand structure creates a new source of pressure: workplace telepressure (Barber and Santuzzi, 2015).

Workplace telepressure is a psychological state in which employees are constantly concerned about urgently responding to work-related ICTs during non-working hours (Grawitch et al., 2017). This phenomenon is caused by job demands and extends beyond the workplace and is more likely to occur in occupations with unclear work-family boundaries (Mazmanian et al., 2013). Considering the flexible working hours of university teachers, the boundaries between work and non-work areas are very vague (Eagan and Garvey, 2015). Therefore, workplace telepressure is more intense. Based on the Job Demands-Resources (JD-R) model, workplace telepressure is a typical kind of job demand (Schaufeli et al., 2009). As a new type of pressure trigger, workplace telepressure will have negative emotional consequences for university teachers. In contemporary times of multitasking, social media, and smartphones, research on mindfulness is increasingly relevant (Decuypere et al., 2020). Therefore, the second purpose of this study is to investigate the effect of workplace telepressure on leader mindfulness and the emotional exhaustion of university teachers.

COR theory suggests that individuals with greater resources are less vulnerable to resource loss and more capable of resource gain (Hobfoll et al., 2018). Some individual characteristics may be viewed as individual personal resources “to the extent that they generally aid stress resistance” (Hobfoll, 1989; p. 517). Among these, this study decided to focus on malleable personal resources rather than stable personality traits as they can be improved *via* proper training, opening the possibility for this study to provide useful practical implications. Mindfulness has been defined as a mental state of openness and acceptance and being present in the moment (Schuh et al., 2019). Mindfulness can be considered an initial resource that helps university teachers obtain leader mindfulness resources more effectively. Furthermore, COR theory also indicates that initial resources can help to reduce resource loss (Hobfoll et al., 2018). In the workplace, self-efficacy in managing negative emotions has been proven to be an important personality trait in easing negative effects induced by work-related stress (Caprara et al., 2013). According to COR theory, personal malleable characteristics represent resources that generally aid stress resistance (Hobfoll, 1989), suggesting that certain malleable characteristics can be regarded as personal coping resources (Sommovigo et al., 2019; Maffoni et al., 2020). Therefore, self-efficacy in managing negative emotions can be regarded as a personal coping mechanism that helps university teachers to prevent resource loss cycles. Workplace telepressure is a characteristic negative emotional event related to work. Taking workplace telepressure as the event node, this study investigates the *ex ante* situational role of university teacher mindfulness in preventing workplace telepressure, and the *ex post* boundary condition of self-efficacy in managing negative emotions to aid in workplace telepressure relief when it does occur. Furthermore, scholars have also suggested that the role of personal resources should be considered in the JD-R model, and that their position in the model should be determined through empirical research (Schaufeli and Taris, 2014). Therefore, the third purpose of this study is to examine the moderating effect of university teacher mindfulness on the relationship between leader mindfulness and workplace telepressure, as well as the moderating effect of self-efficacy in managing negative emotions on the relationship between workplace telepressure and emotional exhaustion in university teachers.

In summary, this study combines COR theory with the JD-R model, and applies a resources crossover effect perspective to examine the crossover effect of leadership resources. In particular, this study examines the interpersonal influence of leader mindfulness on the emotional exhaustion of university teachers. The mediating role of workplace telepressure and the moderating role of university teacher mindfulness and self-efficacy in managing negative emotions are also examined.

## Theoretical Basis and Literature Review

### Theoretical Basis

COR proposes that individuals possess a finite number of resources, like time, knowledge, self-esteem, and conditions like job security or social relationships at work. Individuals strive to obtain, retain, foster, and protect these resources (Hobfoll, 1989). COR theory posits that stress occurs when there is a threat of resource loss, when resources are lost, or when there is a failure to gain resources following significant effort (Hobfoll, 2001). Those with greater initial resources are less vulnerable to resource loss and are more capable of resource gain (Hobfoll, 2001).

The Job Demands-Resources (JD-R) model was first posited by Demerouti et al. (2001) in an attempt to understand the antecedents of employee burnout. The JD-R model proposed two processes for the development of burnout: long-term extreme job demands and a lack of resources (Demerouti et al., 2001). Schaufeli and Bakker (2004) presented a revised version of the JD-R model that included work engagement, in addition to burnout, as part of the motivational process and the health erosion process. Through the health erosion process, sustained effort to meet job demands contributes to produce strain through the exhaustion of energy reserves (Bakker and Demerouti, 2007). The motivational process describes the potential allocation of job resources, including the relief of job demands and the promotion of work engagement (Bakker and Demerouti, 2007). With the development of the JD-R model, scholars suggested that personal resources should be integrated into the JD-R model and the resources’ position in the model should be determined through empirical research (Schaufeli and Taris, 2014).

This study combined COR theory with the JD-R model to suggest research hypotheses. According to the COR theory, leader mindfulness is a kind of job resource, which can directly prevent the loss of university teachers’ resources, i.e., alleviate emotional exhaustion. According to the JD-R model, the provision of job resources can relieve the negative impact of job demands, to prevent further loss of resources (Schaufeli, 2017). It can be inferred that leader mindfulness can alleviate university teachers’ emotional exhaustion by reducing workplace telepressure. Hobfoll (2011) stated that resources do not exist individually but exist in caravans. Resources are likely to emerge from nurturing or supportive social conditions. Therefore, a new line of research should investigate the crossover of resources (Hobfoll et al., 2018). Leader mindfulness and the personal resources of university teachers do not exist individually, and personal resources can exert the initial effect of resources (Hobfoll et al., 2018).

Furthermore, previous studies have shown that job resources moderate the relationship between work engagement and personal resources like self-efficacy, optimism, and organization-based self-esteem (Xanthopoulou et al., 2007). Personal resources like mindfulness regulate the influence of work resources on psychological stress (Grover et al., 2017). Personal resources such as self-efficacy mediate the impact of job resources and challenging work pressures on university teachers’ well-being (Han et al., 2020). However, research on the effectiveness of personal resources in gaining further resources and preventing resource loss is still scarce. In this study, both resource gain and resource loss prevention are considered. Therefore, this study explores the moderating role of the personal resources of university teachers (university teacher mindfulness and self-efficacy in managing negative emotions) in promoting the acquisition of resources (the process of leader mindfulness relieving workplace telepressure) and preventing the further loss of resources (the process of workplace telepressure promoting emotional exhaustion).

### Role of Mindfulness in the Workplace

From the perspective of *intrapersonal* effects, research has identified positive effects of mindfulness in the workplace in three key areas. First, mindfulness has been linked to improved well-being. Several studies have suggested that mindfulness can be considered a personal resource that enables individuals to manage job stressors more effectively (Bergin and Pakenham, 2016; Kaplan et al., 2017), such as role conflict (Montani et al., 2019). Furthermore, mindfulness is linked to reduced emotional exhaustion (Hülsheger et al., 2013), increased resilience (Malinowski and Lim, 2015), and improved sleep quality (Hülsheger et al., 2015). Second, mindfulness is related to positive work attitude and performance. Research has also linked mindfulness to better job performance (Dane and Brummel, 2014), authentic functioning and work engagement (Leroy et al., 2013), extra-role efforts (Krishnakumar and Robinson, 2015), and safety performance (Zhang et al., 2013). Third, research has found that mindfulness has a positive effect on increasing awareness of the limitations of one’s own thinking process. For example, studies suggest that mindfulness improves the quality of decision-making by reducing cognitive biases (Hafenbrack et al., 2014) and enhances ethical decision-making (Karelaia and Reb, 2015). Mindfulness can eliminate stereotypical thinking, and an open, non-judgmental manner facilitates the generation of more creative ideas (Baas et al., 2014).

Although a significant amount of empirical and conceptual work has explored the effects of mindfulness in the workplace, there is a lack of research that examines the *interpersonal* aspects of mindfulness (Good et al., 2016). Only a few studies have investigated the positive influence of leader mindfulness on employees (Schuh et al., 2019); for example, based on the social exchange theory, Reb et al. (2014) explored the alleviation effect of leader mindfulness on employees’ emotional exhaustion. Reb et al. (2019) further explored how leader mindfulness relieved employee stress. However, these studies did not conduct an exploration of internal mechanisms. Based on justice theory, Schuh et al. (2019) explored the internal mechanism of leader procedural justice enactment between leader mindfulness and employee exhaustion. However, this mechanism does not have the motivating force of leader mindfulness resource flow to explain the process of employees’ emotional exhaustion. A previous study indicated that the situational effect of employee mindfulness on the effectiveness of leader mindfulness should be best explored from a bilateral leader-employee perspective (Reb et al., 2019).

The principle of leader mindfulness in the educational context is identical to that in the workplace context, in that it focuses on the current state and perception of employees, and manages subordinates in a compassionate way (Schuh et al., 2019). Mindful leaders are able to provide employees with better emotional support and more emotional resources (Decuypere et al., 2020). Education leaders in schools practice mindfulness by not judging others, being fully present, utilizing emotions and social intelligence, and being sympathetic to others in the organization (Gates and Gilbert, 2016). Mindfulness research in the field of education provides empirical evidence for the theoretical links underlying this current study.

## Hypotheses Development

### Leader Mindfulness and Emotional Exhaustion of University Teachers

Emotional exhaustion denotes a state of extreme mental fatigue and emotional resource exhaustion under pressure (Watkins et al., 2014) and manifests in the excessive loss of individual resources. Based on COR theory, resource supplementation and the prevention of resource loss are essential to alleviating emotional exhaustion (Hobfoll, 2011). Recent research on COR theory states that resources in an organization are subject to the crossover effect of interpersonal flow (Gutermann et al., 2017). As leaders occupy more influential positions in the workplace and control more resources, they can be regarded as credible sources of the outflow of resources (Rogers and Ashforth, 2017). As a source of resources, leader mindfulness can effectively supplement the resources of university teachers, prevent the loss of resources, and alleviate emotional exhaustion.

This process of assisting teachers has three steps. First, a mindful leader can help university teachers consciously separate themselves from the pressures associated with working situations (Good et al., 2016). Research showed that employees who feel more supported in the workplace also score higher in mindfulness tests (Decuypere et al., 2020). Therefore, it can be inferred that the mindfulness of university teachers can be improved with the support of mindful leaders. Mindfulness enables individuals to view stress in a more positive and objective way through the practice of being fully present (Weinstein and Ryan, 2011). University teachers can thereby separate themselves from the pressures associated with working situations (Good et al., 2016), reducing their experience of work-related pressure. Because emotional exhaustion is a stress response caused by exceptional mental exhaustion and extreme fatigue under pressure (Watkins et al., 2014), one can speculate that emotional exhaustion will be relieved when work pressure is reduced.

Second, mindfulness can enable leaders to have more sympathy toward, and have intelligent responses to, university teachers, communicate and interact with other employees effectively, and pay more attention to university teachers’ needs (Reb et al., 2019). Leader mindfulness can thus become a supplement to the resources of university teachers. According to COR theory, individuals who possess greater resources may perceive job stressors as challenging, rather than threatening, conditions (Sommovigo et al., 2019). Having more resources will further reduce the risk of university teacher emotional exhaustion.

Third, combined with the JD-R model, job resources can relieve work pressure (Schaufeli, 2017). Job resources are physical, social, or organizational aspects of the job that are instrumental in achieving work goals (Schaufeli and Taris, 2014). Leader mindfulness can be regarded as a kind of job resource, which can effectively relieve the pressure experienced by university teachers. Emotional exhaustion, as a psychological strain response to work stress (Watkins et al., 2014), can also be relieved. Thus, we formulated the following hypothesis:

H1: Leader mindfulness is negatively related to the emotional exhaustion of university teachers.

### Mediating Effect of Workplace Telepressure

Workplace telepressure is defined as an impulse to concentrate on, and be anxious to respond immediately to, work-related messages sent *via* ICTs (Barber and Santuzzi, 2017). The use of ICTs restricts the autonomy of professionals, as they can be technologically connected to work at all hours of the day and night (Mazmanian et al., 2013). As a type of knowledge professional, the greater autonomy held by university lecturers makes it possible for them to be subjected to more workplace telepressure. Workplace telepressure is a typical working stress caused by job demand (Barber and Santuzzi, 2015). According to the JD-R model, job resources can act as a buffer on restrictions induced by job demand (Bakker and Demerouti, 2007), and the job resources delivered by leader mindfulness can effectively alleviate stress. According to the concept of caravans in COR theory, resources do not exist individually but travel in caravans (Hobfoll, 2011). Personal resources are likely to emerge from nurturing or supportive working conditions (Hobfoll et al., 2018). Therefore, mindful leaders will assist with the cultivation of more personal resources for university teachers, which will help the university teachers cope with workplace telepressure.

Leader mindfulness helps university teachers focus on the present moment instead of problems caused by distraction (Sutcliffe et al., 2016). This can help university teachers to divert their attention from their job during non-working time. Psychologically detaching from work is a helpful way for university teachers to alleviate workplace telepressure (Smit and Barber, 2016).

Mindful leaders have a sympathetic perception of the state of university teachers and cultivate reflective behaviors for them (Gates and Gilbert, 2016). Therefore, mindful leaders are concerned about the feelings of university teachers and will not require university teachers to reply to ICT demands immediately. As mentioned previously, the requirement of an immediate response to asynchronous work-related messages promotes workplace telepressure (Barber and Santuzzi, 2017). Leaders’ mindfulness is reflected in a mindful communication style (Arendt et al., 2019), which is positively related to enhanced communication in school settings (Kearney et al., 2013). High-quality communication will reduce the need for employees to be continuously connected to the workplace through ICTs, thus relieving workplace telepressure (Barber and Santuzzi, 2017). Additionally, leaders report that mindfulness helps them focus on single task instead of “multitasking” (Decuypere et al., 2020). Increased focus on a single task reduces leaders’ distribution of remote tasks during non-working hours, thus reducing the source of workplace telepressure on university teachers.

Finally, mindful leaders who are emotionally and socially intelligent will convey a non-judgmental view to university teachers (Gates and Gilbert, 2016). Stress does not only originate from the event itself but also from an individual’s negative evaluation of the event (Sonnentag and Fritz, 2015). Leader mindfulness promotes university teachers’ adaptability to work-related stressful events (Hülsheger et al., 2015). Therefore, the perceptions of work stress and job demand induced by workplace telepressure will be reduced. Thus, we formulated the second hypothesis:

H2: Leader mindfulness is negatively related to university teachers’ workplace telepressure.

COR theory indicates that resources have a depletion spiral, and the initial loss of resources will cause individuals to experience tension and pressure, which will lead to further loss of resources (Hobfoll, 2011). First, workplace telepressure is a job demand that results in a greater connection to the work environment, even beyond the workplace (Barber and Santuzzi, 2017). As a result, university teachers need to expend additional resources to cope with the pressure (Grawitch et al., 2017). This pressure results in the loss of resources and further increases the risk of emotional exhaustion.

Second, according to COR theory, individuals need to continuously invest in resources to maintain and restore the necessary level of resources needed to cope with work-related stress (Ito and Brotheridge, 2003). Workplace telepressure makes it impossible for university teachers to recover from work-related pressures during non-work time (Barber and Santuzzi, 2017). Being constantly connected with work will reduce psychological detachment (Smit and Barber, 2016), which will impede university teachers from obtaining new physical and mental resources by participating in recovery and leisure activities. Consequently, physical and cognitive resources cannot be supplemented in time, and workplace telepressure will increase the possibility of fatigue and emotional exhaustion.

Third, according to the COR theory, resources can flow interpersonally and in a top-down fashion within a hierarchy, causing crossover effects (Hobfoll et al., 2018). Leader mindfulness can be regarded as a job resources supplement. Combined with the JD-R model, job resources can ease the pressures of work requirements and supplement the loss of personal resources (Hu et al., 2011). Therefore, leader mindfulness can reduce university teachers’ workplace telepressure and further relieve emotional exhaustion. Therefore, we formulated the third hypothesis:

H3: Workplace telepressure mediates the relationship between leader mindfulness and the emotional exhaustion of university teachers.

### Moderating Effect of University Teacher Mindfulness

COR theory indicates that resources exert an initial resources effect, which can reduce resource loss and increase the acquisition of new resources (Hobfoll et al., 2018). As a typical initial resource, individual’s malleable characteristics play an important role in resisting resource loss and improving resource acquisition, all of which help individuals to resist stress (Hobfoll, 2011). Mindfulness, as a mental state of openness, acceptance, and being present in the moment (Schuh et al., 2019), can be considered an initial resource that helps university teachers to more effectively obtain leader mindfulness resources.

A previous study has highlighted the fact that negative attitudes often lead individuals to focus on their internal views, often making them unwilling to accept others’ views (Yip and Schweitzer, 2019). In contrast, university teachers with higher mindfulness will interact with their leaders in a more open and positive manner (Murnieks et al., 2019). Therefore, university teachers can more effectively perceive the information transmitted via leader mindfulness, and the effect of leader mindfulness on reducing workplace telepressure will be more significant.

Another previous study has shown that when the authenticity and intensity of emotional expression between an individual and other people do not match, the individual’s perception of the rationality of others’ emotional expression will be insufficient (Yip and Côté, 2013). Mindful university teachers are open and receptive to their current experiences (Schuh et al., 2019), which will enable them to have the same perceptions and levels of attention as mindfulness leaders. University teachers with a higher level of mindfulness have a stronger understanding of the emotional expressions of mindfulness leaders. Therefore, the effect of leader mindfulness on relieving work telepressure will be enhanced.

Furthermore, according to the COR theory, possessing initial resources enhances the capability of individuals to acquire new resources (Halbesleben et al., 2014). Mindfulness equips leaders with more sympathy and empathy (Good et al., 2016) and consequently, they can engage in higher-quality communication and interactions with university teachers (Arendt et al., 2019). Mindful leaders are also more committed to high-quality social exchanges with university teachers (Reb et al., 2019). As an initial resource, mindfulness helps university teachers obtain the resources transmitted by leader mindfulness. This acquisition of new resources helps prevent resource loss, i.e., the mitigation effect of leader mindfulness on workplace telepressure. Thus, this study formulated the fourth hypothesis:

H4: University teacher mindfulness positively moderates the relationship between leader mindfulness and workplace telepressure.

### Moderating Effect of Self-Efficacy in Managing Negative Emotions

In the workplace, self-efficacy in managing negative emotions has been proven to be an important individual’s malleable characteristic for managing work stress (Caprara et al., 2013). Individuals with high self-efficacy in managing negative emotions can effectively recover from negative emotional states caused by adversities at work (Alessandri et al., 2018). This ease of recovery can facilitate the reflection on the own function of individuals and encourage the acquisition of advantageous experiences (Alessandri et al., 2015).

University teachers who are efficient in managing their negative emotions have strong coping mechanisms and recovery capabilities when facing the impact of workplace telepressure (Caprara et al., 2013). Consequently, the negative impact of workplace telepressure declines. A high level of self-efficacy when managing negative emotions enhances reflexivity and generates advantageous experiences in university teachers (Alessandri et al., 2015). Their assessment of workplace telepressure will be more objective. Therefore, high self-efficacy in managing negative emotions will relieve the effect of workplace telepressure on emotional exhaustion.

Additionally, self-efficacy in managing negative emotions helps university teachers more flexibly address negative experiences (Milioni et al., 2015). Workplace telepressure is a typical negative work experience. A higher level of self-efficacy in managing negative emotions can help university teachers better control their negative emotions, enabling them to be less easily affected by emotional fluctuations when faced with adversity or challenges (Caprara et al., 2013). This contributes to alleviating the shock of negative emotions and relieves the emotional exhaustion caused by workplace telepressure.

Furthermore, according to the COR theory, self-efficacy in managing negative emotions, as an abundant initial resource for university teachers, will reduce the extent of resource loss and prevent teachers from falling into a loss spiral when faced with resource loss (Hobfoll et al., 2018). Therefore, as a kind of resource loss, the impact of workplace telepressure on emotional exhaustion will be reduced. A high level of self-efficacy in managing negative emotions helps university teachers become more adaptive and maintain basic emotional stability (Alessandri et al., 2018). Therefore, workplace telepressure, as an example of resource loss, will have less impact on the spiral of resource depletion caused by emotional exhaustion. As a result, this study presents a fifth hypothesis:

H5: Self-efficacy in managing negative emotions negatively moderates the relationship between workplace telepressure and emotional exhaustion of university teachers.

**FIGURE 1:**
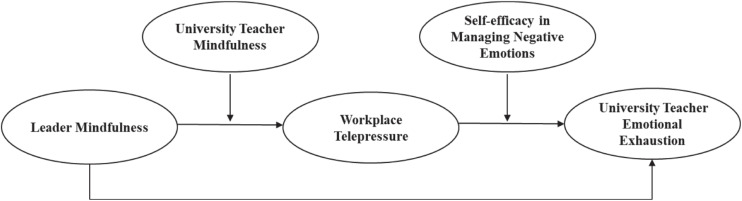
Theoretical model.

The conceptual model is shown in Figure 1.

## Methods

### Sample and Procedure

Questionnaires were distributed to public university teachers, excluding full-time administrative staff, in first-tier cities such as Beijing, Shanghai, Shenzhen, and Guangdong. A total of 43 universities were randomly selected for investigation. To reduce common method bias, this study adopted a time-lagged research design lasting for 6 months. An online questionnaire survey was adopted, and questionnaires were sent to university leaders and teachers, who were informed of the voluntary nature of participation in the survey and assured of the confidentiality of their responses. University teachers were requested to take part in three surveys lasting a total of 6 months. Participants could withdraw at any stage of the study without any negative consequences.

At the baseline time point in October 2019, we asked university leaders to complete an online survey on measuring leader mindfulness and demographic variables including gender, age, and tenure in their current position. We also asked university leaders to provide us with the contact information of at least five direct subordinates, who were university teachers. We contacted these subordinates and asked them to complete an online survey on university teacher mindfulness and self-efficacy in managing negative emotions and demographic variables including gender, age, tenure in their current position, and weekly working hours. The last four digits of the participating university teachers’ mobile phone numbers were used as the matching codes to track the questionnaire replies accurately. A total of 546 paired questionnaires were distributed, 489 were recovered, and 462 were identified as valid. In phase 2, about 3 months later, we contacted the university teachers and asked them to complete the online survey on workplace telepressure. A total of 412 questionnaires were collected, 408 of which were valid. During phase 3, an additional three months later, we asked university teachers to rate their emotional exhaustion. A total of 397 questionnaires were collected, 388 of which were valid. We introduced reverse questions to reduce common method bias and rejected questionnaires with an obvious trend to the answers, such as choosing the same option for all questions. Additionally, according to previous research, a questionnaire with a response rate below 30% is considered an invalid questionnaire (Newman, 2014). For those questionnaires with a small amount of missing data, we used the mean value instead.

The final effective response rate was 71.06%. In the sample of university leaders, 69.38% were male, 30.63% were female, the average age was 43.67 years (SD = 5.02), and the average tenure in the current position was 13.38 years (SD = 6.77). In the sample of university teachers, 42.17% were male, 57.83% were female, the average age was 40.68 years (SD = 7.53), the average tenure in the current position was 8.46 years (SD = 5.72), and the average weekly working hours were 48.37 h (SD = 8.56).

### Measures

Through a systematic review of the relevant literature on mindfulness, workplace telepressure, self-efficacy in managing negative emotions, and emotional exhaustion, this study collated the measurement scales of the relevant constructs. A preliminary English version of the survey instrument was derived from a thorough literature review of the relevant constructs and measures. Following the procedure set by Brislin (1980), a bilingual researcher translated all English items into Chinese. Another researcher translated the items back into English. A comparison showed high levels of translation accuracy. We presented all items in Mandarin Chinese.

#### Independent Variable

Leader mindfulness was measured based on an established scale derived from Brown and Ryan (2003). This scale has been frequently used in earlier studies (e.g., Hülsheger et al., 2013; Roche et al., 2014). In accordance with previous research (Grant et al., 2009; Schuh et al., 2019), the present study used the five items with the highest factor loadings. A sample item is “I rush through activities without being really attentive to them in the management,” and all items were reverse coded. Items were rated on a 7-point scale from 1 = *almost always* to 7 = *almost never* (Cronbach’s α = 0.834).

#### Mediation Variable

Workplace telepressure was measured with a six-item scale derived from Barber and Santuzzi (2015). A sample item is “when I receive work information, it is difficult for me to focus on other things.” Items were rated on a 7-point scale from 1 = *strongly disagree* to 7 = *strongly agree* (Cronbach’s α = 0.904).

#### Moderation Variable

University teacher mindfulness was measured based on an established scale derived from Brown and Ryan (2003). In accordance with previous research (Grant et al., 2009; Schuh et al., 2019), the present study used the five items with the highest factor loadings. A sample item is “I rush through activities without being really attentive to them,” and all items were reverse coded. Items were rated on a 7-point scale from 1 = *almost always* to 7 = *almost never* (Cronbach’s α = 0.830). Self-efficacy in managing negative emotions was measured with a six-item scale derived from Alessandri et al. (2018). A sample item is “I can keep calm during stressful and straining situations.” Items were rated on a 7-point scale from 1 = *strongly disagree* to 7 = *strongly agree* (Cronbach’s α = 0.889).

#### Dependent Variable

Emotional exhaustion was measured with a three-item scale derived from Watkins et al. (2014). A sample item is “I feel emotionally drained from my work.” Items were rated on a 7-point scale from 1 = *strongly disagree* to 7 = *strongly agree* (Cronbach’s α = 0.912).

#### Control Variable

Based on previous research, age, gender, tenure in their current position, and weekly working hours were controlled, since each of these measures could influence emotional exhaustion (Moen et al., 2013; Charoensukmongkol, 2016; Murnieks et al., 2019). Age is correlated with mindfulness, because as people age they become more mindful (Hohaus and Spark, 2013; Grover et al., 2017). To avoid spurious findings, we controlled for the age of university teachers in the regression equations. Tenure in their current position and weekly working hours are positively correlated with emotional exhaustion (Charoensukmongkol, 2016; Murnieks et al., 2019). Gender was measured as a dummy variable, where male was coded 1 and female was coded 0. Age and tenure in their current position were measured in years, and weekly working hours were measured in hours.

### Common Method Bias

This study primarily used SPSS 22.0 (IBM Corp., Armonk, NY, United States) and LISREL 8.80 (Scientific Software International, Inc., Lincolnwood, IL, United States) for data analysis and hypothesis testing. Harman’s single-factor test was adopted to estimate the common method bias (Podsako et al., 2003). The results indicated that the cumulative explanation variation degree of the five factors was 78.40%, and the explanation variation degree of the first factor was 21.179%. Thus, no single factor had particularly significant explanatory power, indicating that no homologous error existed. The fitting degree of the single factor model was poor (χ^2^ = 1,550.769, *df* = 152, χ^2^/*df* = 10.202, Root Mean Square Error of Approximation (RMSEA) = 0.127, Comparative Fit Index (CFI) = 0.780, Incremental Fit Index (IFI) = 0.781, Normed Fit Index (NFI) = 0.763). Therefore, no significant common method bias problem was found. As an additional testing measure, this study added an unmeasured latent factor to the measurement model. If such a method factor existed, the model would have a better fit compared with the model without this factor (Podsakoff et al., 2012). The addition of a common method factor to the five-factor model did not improve the fit: the changes in the fit indexes (Δχ^2^ = 219.320, Δ*df* = 22.000, Δχ^2^/*df* = 0.866, ΔRMSEA = 0.013, ΔNFI = 0.039, ΔCFI = 0.017, ΔIFI = 0.037, △ Goodness-of-Fit Index (GFI) = 0.040) were clearly below the recommended values to indicate a more parsimonious model (i.e., an increase of at least 0.02 in CFI, Vandenberg and Lance, 2000; a decrease of at least 0.015 in RMSEA, Chen, 2007). In addition, the mean of the squares of the standardized factor loadings of the different items on the unmeasured latent method factor is 0.18 (< 0.25), which indicated that common method bias does not appear to have a substantial impact on the present study. Although adding this additional latent factor did result in a slightly better fit, it does not rule out the possibility that there may be merit to our results (Conway and Lance, 2010), especially regarding interactions (Siemsen et al., 2010).

### Power Analysis, Reliability, and Validity

Power analysis for a multiple regression analysis with eight predictors was conducted using G^∗^Power (Faul et al., 2009) to determine whether the sample had a sufficient size using an alpha of 0.05, a power of 0.95, and a medium effect size. Results indicated that a total sample size of 160 was required, suggesting that our sample size of 388 was adequate.

For the reliability test, as shown in Table 1, the Cronbach’s α of each construct was found to exceed the cutoff value of 0.70. Composite reliability (CR) estimation indicates good reliability ranging from 0.841 to 0.928, which exceeds the recommended threshold of 0.7.

**TABLE 1:** Correlation coefficients between each construct.

Construct	Skewness	Kurtosis	CR	AVE	√AVE	1	2	3	4	5
Leader mindfulness	−0.499	0.739	0.896	0.653	0.808	**0.834**				
Workplace telepressure	0.368	0.101	0.918	0.652	0.807	−0.448*	**0.904**			
University teacher mindfulness	−0.220	0.007	0.841	0.607	0.779	0.440*	−0.417**	**0.830**		
Self-efficacy in managing negative emotions	−0.677	0.710	0.908	0.623	0.789	0.509**	−0.327*	0.302*	**0.889**	
Emotional exhaustion	0.584	1.144	0.928	0.812	0.901	−0.486*	0.381*	−0.399**	−0.495**	**0.912**

The diagonal numbers formatted in bold indicate Cronbach’s α, √AVE indicates the square root of AVE. χ^2^ = 686.838, df = 199, χ^2^/df = 3.451, RMSEA = 0.069, SRME = 0.048, CFI = 0.913, GFI = 0.882, AGFI = 0.850, TFI = 0.883, IFI = 0.914, NFI = 0.883. *p < 0.05; **p < 0.01.

Regarding the validity of each construct, as shown in Table 1, the average variance extraction (AVE) of each construct ranges from 0.607 to 0.812, exceeding the standard value of 0.5 and indicating good convergent validity. As shown in Table 1, the square root of the AVE of each variable exceeds the correlation coefficients between the latent variables, further suggesting that the scale had good discriminant validity. Additionally, as shown in Table 2, the results of the model fit test using confirmatory factor analysis (CFA) indicate that our hypothesized 5-factor model fit the data best (χ^2^ = 686.838, *df* = 199, χ^2^/*df* = 3.451, RMSEA = 0.069, SRME = 0.048, CFI = 0.913, GFI = 0.882, AGFI = 0.850, TFI = 0.883, IFI = 0.914, NFI = 0.883), providing support for the hypothesized 5-factor model and the distinctiveness of the variables in this study.

**TABLE 2:** Comparisons of measurement models.

Factor structure	χ^2^	*df*	χ^2^/*df*	RMSEA	SRME	CFI	GFI	AGFI	NFI	TLI	IFI
1-factor model	1,328.468	209	6.356	0.102	0.070	0.801	0.769	0.721	0.773	0.780	0.802
2-factor model	1,273.487	208	6.123	0.100	0.069	0.810	0.779	0.731	0.782	0.789	0.811
3-factor model	1,129.926	206	5.485	0.094	0.076	0.836	0.802	0.757	0.807	0.816	0.836
4-factor model	1,103.347	203	5.435	0.093	0.065	0.840	0.803	0.754	0.811	0.818	0.841
5-factor model	686.838	199	3.451	0.069	0.048	0.913	0.882	0.850	0.883	0.899	0.914

*One-factor model (leader mindfulness + workplace telepressure + university teacher mindfulness + self-efficacy in managing negative emotions + emotional exhaustion). Two-factor model (leader mindfulness + workplace telepressure + university teacher mindfulness + self-efficacy in managing negative emotions + emotional exhaustion). Three-factor model (leader mindfulness + university teacher mindfulness, workplace telepressure + self-efficacy in managing negative emotions, emotional exhaustion). Four-factor model (leader mindfulness, university teacher mindfulness + self-efficacy in managing negative emotions, workplace telepressure, emotional exhaustion). Five-factor model (leader mindfulness, workplace telepressure, university teacher mindfulness, self-efficacy in managing negative emotions, emotional exhaustion).*

### Multivariate Normal Test

**FIGURE 2:**
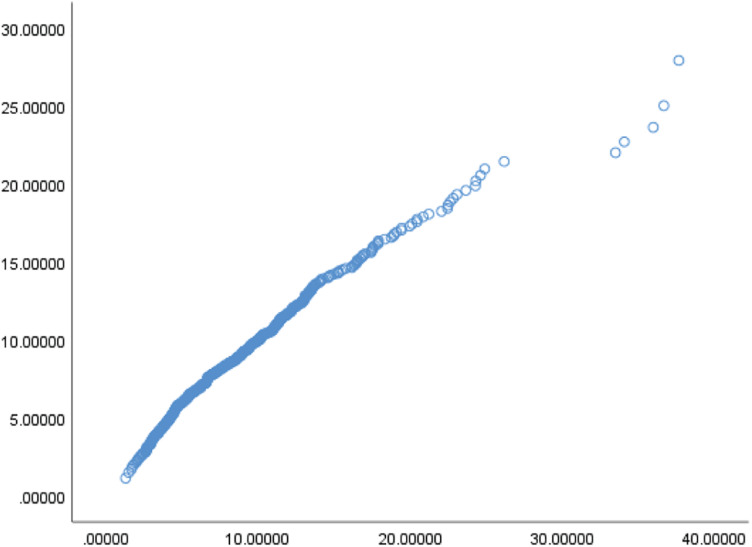
Chi-square and Mahalanobis distance plot.

Kim (2013) states that if the —skewness— > 2 or —kurtosis— > 7, the data did not form a normal distribution. Table 1 shows the skewness and kurtosis of each variable. The results indicate that the sample presents a normal distribution. Additionally, if the variables form a multivariate normal distribution, the chi-square and Mahalanobis distance plot will form a straight line (Nor, 2015). Figure 2 shows that the variables of this sample form a multivariate normal distribution.

## Hypotheses Testing and Results

In this study, multiple regression was used to test each hypothesis. Four regression models were constructed to test the direct effects of leader mindfulness and the mediating effects of workplace telepressure. Two groups of stepwise regression models were constructed to test the moderating effects of university teacher mindfulness and self-efficacy in managing negative emotions. A multicollinearity test was also conducted in this study. The variance inflation factors (VIFs) of the regression models were significantly lower than the critical value of 10, and the tolerances of the reciprocals of the regression models were also close to 1, signifying that the results had not been affected by a multicollinearity issue. Additionally, the Durbin-Watson (DW) values of the autocorrelation test results of all the regression models were between 1.5 and 2. Values close to 2 indicate no sequence correlation problem in a regression model. Therefore, the empirical test results of this study were scientific and reliable.

### The Demographic Variables Effect and the Direct Effect of Leader Mindfulness

As shown in Table 3, the results of M1 showed that tenure and hours worked were positively related to university teacher emotional exhaustion. With the variables of gender, age, tenure, and hours worked being controlled, the results of M2 showed that leader mindfulness had a significant negative effect on the emotional exhaustion of university teachers (β = −0.597, *p* < 0.001). Thus, H1 was verified.

**TABLE 3:** Test results of direct and mediating effects.

Variable	Emotional exhaustion		Workplace telepressure	Emotional exhaustion
	M1	M2	M3	M4
Gender	0.007	−0.017	−0.121*	0.001
Age	−0.017	−0.014	0.025	−0.017
Tenure	0.062*	−0.006	−0.051	0.001
Hours worked	0.092*	0.021	0.001	0.021
Leader mindfulness		−0.597***	−0.760***	−0.472***
Workplace telepressure				0.145**
*R*^2^	0.690	0.504	0.400	0.527
Δ*R*^2^	0.690	0.435	0.376	0.023
*F*	10.465***	114.832***	75.246***	104.878***
DW	1.936	1.985	1.987	2.031

n = 388; *p < 0.05; **p < 0.01; ***p < 0.001.

### Mediating Effects of Workplace Telepressure

Regarding the mediating effect of workplace telepressure, as shown in Table 3, the results of regression model M2 (β = −0.597, *p* < 0.001) indicate that leader mindfulness had a significant negative effect on the emotional exhaustion of university teachers. The results of regression model M3 (β = −0.760, *p* < 0.001) indicate that leader mindfulness had a significant negative effect on workplace telepressure. H2 was therefore verified.

As the mediating variables of workplace telepressure were controlled, the results of regression model M4 indicate that the effects of leader mindfulness on university teacher emotional exhaustion remain significant (β = −0.472, *p* < 0.001), and workplace telepressure also had a significant effect (β = 0.145, *p* < 0.001) on university teacher emotional exhaustion. Therefore, workplace telepressure had a partially mediating effect between leader mindfulness and the emotional exhaustion of university teachers, which supports H3.

This study used the PROCESS macro of SPSS (template 4) to further verify the mediating effect of workplace telepressure and used the bootstrapping test to obtain the indirect effect value. As shown in Table 4, the indirect effect of leader mindfulness on the emotional exhaustion of university teachers through workplace telepressure was −0.120, with a standard error of 0.048, and a confidence interval of [−0.213, −0.023]. After controlling the mediating variable of workplace telepressure, the direct effect of leader mindfulness on university teachers’ emotional exhaustion was −0.592, the standard error was 0.050, and the confidence interval was [−0.680, −0.493]. Since zero was not included in the confidence intervals of these effects, workplace telepressure exerts a significant mediating role between leader mindfulness and the emotional exhaustion of university teachers. This result further supports H3.

**TABLE 4:** Bootstrapping test of the mediating effect.

Mediator	Effect	Effect size	Standard error	95% confidence interval	
				Minimum	Maximum
Workplace tele pressure	Indirect effect	−0.120	0.048	−0.213	−0.023
	Direct effect	−0.592	0.050	−0.680	−0.493
	Total effect	−0.712	0.034	−0.778	−0.646

### Moderating Effect of University Teacher Mindfulness

**FIGURE 3:**
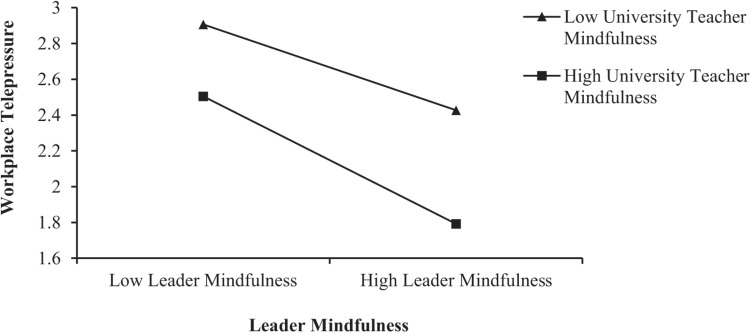
Moderating effect of university teacher mindfulness.

Before testing the moderating effect, to reduce the problem of multicollinearity, leader mindfulness and university teacher mindfulness were centralized. An interaction term was then constructed between leader mindfulness and university teacher mindfulness. As shown in Table 5, the results of the three stepwise regression equations of Model 5, Model 6, and Model 7 show that the interaction term had a significant positive impact on workplace telepressure (β = −0.086, *p* < 0.01). The explanatory power of Model 7 was also significantly enhanced (Δ*R*^2^ = 0.006, thus obeying the *F* distribution). As shown in Table 6, since none of the above confidence intervals contain 0, regardless of whether the university teacher mindfulness levels are low, average, or high, the effect of leader mindfulness on workplace telepressure is significant. The conditional effect analysis indicates that with the increase of university teacher mindfulness, the relieving effect of leader mindfulness on workplace telepressure becomes stronger. Additionally, as shown in Figure 3, compared with low mindfulness levels in university teachers, when university teacher mindfulness was high, leader mindfulness had a stronger effect on reducing workplace telepressure. Therefore, university teacher mindfulness had a positive moderating effect on the relationship between leader mindfulness and workplace telepressure, which supports H4.

**TABLE 5:** Test of the moderating effect.

Variable	Workplace telepressure			Emotional exhaustion		
	Model 5	Model 6	Model 7	Model 8	Model 9	Model 10
Gender	0.007	0.004	0.003	−0.006	0.002	−0.001
Age	−0.001	−0.010	−0.013	−0.006	−0.006	−0.006
Tenure	−0.046	−0.053*	−0.056*	0.064*	0.064*	0.060*
Hours worked	0.002	0.027	0.030*	−0.044*	−0.035*	−0.035*
Z LM	−0.760***	−0.559***	−0.578***			
Z UTM		−0.329***	−0.329***			
Z LM * Z UTM			−0.086**			
Z WT				0.505***	0.388***	0.389***
Z SMNE					−0.199***	−0.210***
Z WT * Z SMNE						−0.051*
*R*^2^	0.562	0.625	0.631	0.358	0.403	0.406
△*R*^2^	0.562	0.063	0.006	0.358	0.045	0.003
F	129.778***	139.972***	122.659***	56.405***	56.750***	49.080***
DW	2.068			1.976		

n = 388. LM, Leader mindfulness; UTM, university teacher mindfulness; WT, workplace telepressure; SMNE, self-efficacy in managing negative emotions. *p < 0.05; **p < 0.01; ***p < 0.001.

**TABLE 6:** Bootstrapping test of the moderating effect.

Moderator	Effect	Effect size	Standard error	95% confidence interval	
				Minimum	Maximum
University teacher mindfulness	Low	−0.5045	0.0490	−0.6007	−0.4084
	Mean	−0.5624	0.0465	−0.6537	−0.4712
	High	−0.6203	0.0548	−0.728	−0.5127
Self-efficacy in managing negative emotions	Low	0.4531	0.0457	0.3634	0.5428
	Mean	0.406	0.0373	0.3328	0.4792
	High	0.3589	0.0451	0.2703	0.4474

### Moderating Effect of Self-Efficacy in Managing Negative Emotions

**FIGURE 4:**
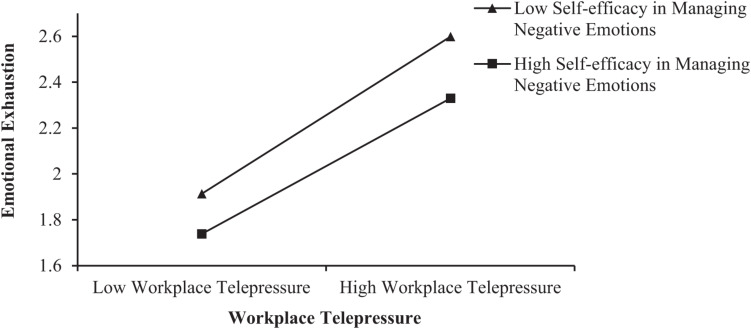
Moderating effect of self-efficacy in managing negative emotions.

Workplace telepressure and self-efficacy in managing negative emotions were centralized. An interaction term was then constructed between workplace telepressure and self-efficacy in managing negative emotions. As shown in Table 5, the results of the three stepwise regression equations of Model 8, Model 9, and Model 10 showed that the interaction term exerted a significant negative impact on emotional exhaustion (β = −0.051, *p* < 0.05). The explanatory power of Model 10 was also significantly enhanced (Δ*R*^2^ = 0.003, thus obeying the *F* distribution). As shown in Table 6, since none of the above confidence intervals contain 0, regardless of whether the self-efficacy in managing negative emotions is low, average, or high, the effect of workplace telepressure on the emotional exhaustion of university teachers is significant. The conditional effect analysis indicates that with the increase of university teachers’ self-efficacy in managing their negative emotions, the positive effect of workplace telepressure on emotional exhaustion becomes weaker. Additionally, as shown in Figure 4, when the self-efficacy in managing negative emotions was high, workplace telepressure had a less significant effect on the emotional exhaustion of university teachers. Therefore, self-efficacy in managing negative emotions exerted a negative moderating effect on the relationship between workplace telepressure and the emotional exhaustion of university teachers. Thus, H5 was supported.

## Discussion

### Theoretical Contributions

Leader mindfulness significantly reduces emotional exhaustion in university teachers. First, this conclusion is consistent with previous research, which has found that leader mindfulness can alleviate employee emotional exhaustion (Schuh et al., 2019). This conclusion, reached in the field of organizational management research, is now extended to educational situations, which proves the significance of leader mindfulness and provides empirical evidence for the effectiveness of mindful educational leadership. Second, previous research on the process of leader mindfulness was primarily based on self-determination theory (Arendt et al., 2019), social exchange theory (Reb et al., 2019), affective event theory (Ruben and Gigliotti, 2016), and justice theory (Schuh et al., 2019). However, the influence of leader mindfulness on subordinates has not been explored from a COR theory perspective. Utilizing the COR theory, this study provides a novel theoretical perspective for studying leader mindfulness. Third, scholars have proposed that the interpersonal transmission process of resources should be investigated by applying a resources crossover effect perspective (Hobfoll et al., 2018). Moreover, researchers have also called for more investigation into the interpersonal effects of leader mindfulness among employees (Reb et al., 2019). To some extent, this study responded to these appeals and investigated the buffering mechanism of university leaders, as a source of outflow of resources, on the loss of university teachers’ resources. Thus, this study contributes to the growing literature on workplace mindfulness.

Workplace telepressure mediates the relationship between leader mindfulness and the emotional exhaustion of university teachers. First, workplace telepressure is a new source of pressure, generated by the continuous development of ICTs (Barber and Santuzzi, 2017). Consequently, the boundaries between university teachers’ work and non-work areas have become very vague (Eagan and Garvey, 2015). Therefore, workplace telepressure is experienced as more intense. Workplace telepressure provides a new perspective for the mechanism underlying the interaction between leader mindfulness and university teacher emotional exhaustion. Second, a previous study has called for more study of the interaction between job resources and demands from the perspective of the JD-R model (Schaufeli, 2017). The conclusions of the present study confirm that job resources can promote the negative impact of high-level job demands. In other words, leader mindfulness can be used as a job resource to reduce workplace telepressure, thus relieving the emotional exhaustion of university teachers. Third, previous studies focused on individuals’ efforts to invest in their own resources (Hobfoll, 2011), while investment in others’ resources has not been considered. The present study used leader mindfulness as a resource outflow source to test its resource investment effect on university teachers. This kind of leadership-oriented resource investment helps university teachers to recover their resources and prevent resource loss. This conclusion extends the epistemology of the COR theory and assesses the mechanism of leader mindfulness by applying a resources perspective.

University teacher mindfulness and self-efficacy in managing negative emotions are situational factors for the improvement of the effectiveness of leader mindfulness. First, Schaufeli and Taris (2014) proposed that personal resources should be considered in the JD-R model and their position in the model should be further determined through empirical research. The current study regarded mindfulness and self-efficacy in managing negative emotions as personal resources of university teachers and determined their situational roles in the JD-R model. Second, based on the COR theory (Hobfoll et al., 2018), the present study verified the initial resources effects of university teacher mindfulness and self-efficacy in managing negative emotions, while determining that their mechanisms were different. Mindfulness increased the possibility of acquiring new resources, while self-efficacy in managing negative emotions reduced individual resource loss and prevented further loss of resources. Mindfulness is the preceding situational condition of workplace telepressure prevention, and self-efficacy in managing negative emotions is the subsequent boundary condition of workplace telepressure relief. Third, in previous studies, the COR theory used personal efforts to acquire, maintain, and protect resources as basic assumptions (Hobfoll, 2011). The present study overcame the “individual participant” perspective and, based on the resources crossover effect as well as from the bilateral perspective of the leader/university teacher, identified the situational conditions for the flow of resources from leaders. Starting from personal’s malleable characteristics, the present study showed that personal resources, which involve university teacher mindfulness and self-efficacy in managing negative emotions, can promote the path of job resources, in this case leader mindfulness, to reduce emotional exhaustion by relieving job demands, which in this study were remote work-related pressures.

### Managerial Implications

Universities will benefit from focusing on, and investing in, the mindfulness of both their leaders and their teachers. Prior studies have suggested that mindfulness can be taught through short mindfulness interventions or mindfulness training sessions, involving instructions that direct people’s attention to the here and now (Long and Christian, 2015; Slutsky et al., 2019). Other previous studies have shown that such interventions and training can have pronounced positive effects on participants’ emotions and actions (Hafenbrack et al., 2014) and that these effects are moderately long-lasting (Hülsheger et al., 2013). Therefore, universities should actively implement college-based mindfulness intervention measures (Roeser et al., 2013). School-based mindfulness interventions can be defined as the adjustment of mindfulness to consciousness and attention, applied to the campus environment. Additionally, professional projects or courses can help leaders improve the effectiveness of their management (Abenavoli et al., 2013). With regard to leader mindfulness, universities can conduct training programs on mindful leadership, tailored to the tertiary education context (for instance, Reb et al., 2019). Moreover, mindful behavior could be established as a selection and performance criterion for leadership positions. Regarding university teacher mindfulness, a broad range of mindfulness programs can be considered, such as mindfulness-based stress reduction or short smartphone-based guided meditation (Walsh and Arnold, 2018).

Furthermore, the benefits provided by ICTs should be viewed as a mixed blessing. ICTs make employees more accessible during non-work hours (Ou et al., 2013), which can make it challenging for employees to detach from work during non-working hours, which in turn can interfere with their domestic life (Derks et al., 2014). Therefore, leaders should formulate rules to specify the usage time and norms of ICTs. Leaders can use event times and calendar sharing as references and clarify that university teachers need to collectively participate in a certain project or deal with specific tasks centrally at a particular time. In other instances, ICTs should not be used to arrange work. University teachers should consciously separate themselves from their jobs to protect their non-working hours from work interference, promote the recovery of their own resources, and prevent falling into a resource loss spiral.

Additionally, university teachers should improve their emotional management and control capabilities when faced with negative emotions. In practice, empirically validated strategies for the development of self-efficacy in managing negative emotions include training programs based on reflective learning (Dacre-Pool and Qualter, 2012) and expressive writing (Kirk et al., 2011). Leaders can conduct self-efficacy counseling for university teachers through these training programs to enhance their psychological flexibility and resilience. These skills will enrich the initial resources of university teachers, improve their capabilities to acquire new resources and resist resource loss, and thus promote occupational health and enable more sustainable development.

### Limitations and Future Research

Despite its value, this study has a number of limitations. First, this study relied solely on self-report measures. The non-experimental nature of the study design does not enable causal inferences. Future research should integrate self-report measures with qualitative data and objective indicators or use an experimental approach to overcome this limitation. Second, this study regards leader mindfulness as a perceived resource at the individual level. New trends in the area of leader mindfulness involve multilevel research into the impact and coherence of mindfulness at various levels in an organization, as well as research into collective mindfulness (Sutcliffe et al., 2016; Decuypere et al., 2020). Future research may explore and enrich the theoretical model of emotional exhaustion from different levels by conducting crosslayer research at the organizational level. Third, this study was conducted in China, and thus it may be challenging to generalize the results to other cultural contexts. Replications of this study should be conducted in other countries. Finally, this study did not directly measure the mechanism that might account for the way in which crossover effects explain how leaders’ mindfulness might be transmitted to their subordinates. Additionally, the results suggested that workplace telepressure is not the only mediating variable between leader mindfulness and emotional exhaustion, and that there might be other complementary mediating variables. Future studies should introduce other mediation variables to enrich the research model and to further develop research on the transmission mechanism of leader mindfulness.

## Conclusion

Drawing on COR theory, integrated with the JD-R model, this study investigated the interpersonal aspects of leader mindfulness. The results indicate the buffering mechanism of university leaders as an outflow of resources on the loss of university teachers’ resources. Workplace telepressure, as a new source of job-based pressure, provides a novel perspective on the mechanism underlying the relationship between leader mindfulness and university teachers’ emotional exhaustion. Additionally, university teacher mindfulness is the preceding situational condition of workplace telepressure prevention, thereby promoting the gain of resources, and self-efficacy in managing negative emotions is the subsequent boundary condition of workplace telepressure relief, preventing cycles of resource loss. Starting from the bilateral personal exploitable traits of leaders and university teachers, this study identified the situational conditions for the effectiveness of resource crossover. This study contributes both to the growing literature on workplace mindfulness, while also offering suggestions for this mindfulness in practice.

## Data Availability Statement

The raw data supporting the conclusions of this article can be provided by the corresponding author on reasonable request, without undue reservation.

## Ethics Statement

According to the stipulations of local legislation and institutional requirements, there is no need for ethical review and approval for questionnaire research on human participants. Written informed consent from the participants was not required to participate in this questionnaire survey in accordance with national legislation and institutional requirements. Guarantees of confidentiality and anonymity and the voluntary nature of participation were provided to respondents to reduce respondent anxiety or answers based on social desirability.

## Author Contributions

BL and QL: conceptualization and writing — review and editing. BL and ZZ: data curation, methodology, and writing — original draft. BL: project administration and funding acquisition. All authors contributed to the article and approved the submitted version.

## Conflict of Interest

The authors declare that the research was conducted in the absence of any commercial or financial relationships that could be construed as a potential conflict of interest.
